# Risk perceptions of COVID-19, vocational identity, and employment aspirations of Chinese aviation students: a structural equation modeling approach

**DOI:** 10.1186/s12889-023-17144-y

**Published:** 2023-11-06

**Authors:** Hongyao Qin, Yong Tang

**Affiliations:** 1 School of Broadcasting and Hosting, Sichuan Film and Television University, Chengdu, Sichuan P.R. China; 2 College of Tourism and Urban-rural Planning, Chengdu University of Technology, Chengdu, Sichuan P.R. China

**Keywords:** COVID-19, Risk perception, Vocational identity, Employment aspiration, Aviation students, Structural equation modeling, China

## Abstract

**Background:**

The COVID-19 pandemic has wreaked havoc on the aviation and education sectors in China. This study examined the relationships between risk perceptions of the pandemic, vocational identity, and employment aspirations of Chinese aviation students.

**Methods:**

The study used a convenience sampling approach to collect data (n = 276 respondents) from August 2 to 8, 2022. An online survey was sent via WeChat and QQ to Chinese students majoring in aviation service management who were under lockdown at six Chinese schools.

**Results:**

In spite of the strong support for the stringent COVID policies and full awareness of infection risk and protective measures, respondents were worried about the current unstable situation and felt fear for its severity and long-lasting symptoms. The casual path from career commitment to employment aspiration was supported, but high risk perceptions of the pandemic failed to have any psychological effect on the two constructs of vocational identity and employment aspirations.

**Conclusions:**

The findings not only demonstrate the power of career commitment on employment aspirations but also reveal that a relatively high self-assessment of career proficiency may not necessarily lead to a clear career aspiration, possibly due to poor risk communication and insufficient career planning guidance. Thus, Chinese aviation students should improve their career proficiency and commitment, broaden their career options and adaptability, and have a clear career plan, in order to be well prepared for the fierce job market that will face the next wave of the ongoing pandemic.

## Introduction

The COVID-19 pandemic is an emerging, rapidly evolving situation that raises a public health emergency of international concern [1]. To address this concern, China has taken the stringent approach of the “Dynamic Zero COVID Strategy”, which has kept infected cases relatively low [2], but it has also exacted a high social and economic cost in metropolitan areas like Shanghai, Shenzhen, and Hangzhou [3–6]. Equally vulnerable to the pandemics is China’s aviation industry [7–9]. For the past three years, China has implemented a set of strict policies to control the flux of potential virus carriers on cross-border flights [10–12]. Under such a scenario, airline workers have still been suffering risks of job instability and virus infection since the outbreak of the pandemic [13].

Despite the recent recovery of scheduled air transport seats around the world [14], a more pressing concern is the on-going pandemic that has posed considerable challenges to the education sector [15], in particular the havoc it has wreaked on the current tertiary aviation education and future careers of its graduates [16]. According to statistics released by the Civil Aviation Administration of China (CAAC), the number of Chinese students enrolled in aviation schools has been steadily declining for the past three years [17]. As a result, the situation that the aviation industry is in a recession with declining enrollment in aviation schools provokes a very haunting question: In response to the risk perceptions of the pandemic, how do Chinese aviation students view their vocational identity and employment aspirations?

Considerable interests from medical and psychological perspectives as well as their intersections have investigated infectious diseases varying enormously from the Bird flu and the Chikungunya, through the Ebola and the Malaria, to the MERS and the SARS [18–20]. Whereas the COVID-19 pandemic has transformed the scientific endeavour of public health in many ways, giving its greater prominence and recognition [21], recent scholarship on public health has focused on the wide-spread psychological and health effects that the pandemic is having on college students around the world, such as nursing and medical students, which highlights the need to foster students’ public health competency, and safely involve students as non-frontline workers in public health emergency responses for their mental wellbeing [22–24]. Yet, there are still many gaps in our emerging understanding of Chinese aviation students’ risk perceptions of the pandemic, which has been defined in this study as “the subjective assessment of the probability and severity of adverse consequences from the COVID-19 pandemic”. A more relevant question is how Chinese aviation students view vocational identity and employment aspirations amidst the stringent Zero COVID policies. These quests have major global health implications for career planning educators, frontline health professionals, and travel medicine practitioners, health communication researchers, and healthcare educators.

To address these urgent needs, this study seeks to investigate the risk perceptions, vocational identity, and employment aspirations of Chinese aviation students amidst the stringent Zero COVID policies. Of particular interest are the relationships between risk perceptions of the pandemic, vocational identity, and employment aspirations. This study will contribute to the debate revolving around the post-pandemic aviation market recovery and long-term career goals of aviation students facing the continuing threat of the pandemic [25, 26].

## Theoretical context

Recent research on risk perceptions has highlighted the wide range of psychological and behavioural responses to the pandemic around the world [27, 28]. It is generally true that psychological flexibility is significantly associated with COVID-19 burnout in Chinese college students, and this link is mediated by perceived COVID-19 stress [29]. While the resulting pandemic has posed severe, distressing, physical, and psychological challenges to Chinese students under lockdown, one issue that has received less attention is how Chinese aviation students respond to the on-going pandemic [30–32]. Of these issues, the focus here is to look at how they view their vocational identity and employment aspirations in response to the pandemic.

According to the knowledge theory, people would perceive the pandemic as dangerous because they knew it to be dangerous [33]. In this respect, college students would have worried about the current situation only when they were fully aware of the infection risk and had sufficient knowledge of the COVID control policies [34]. Yet, empirical evidence also suggests that infection risk does not seem to affect cognition in college students [35], when they possess insufficient COVID-19 knowledge but high infection risk perceptions [36]. Thus, the hypothesis is that the risk perception of the pandemic would neither have an effect on the employment aspirations of Chinese aviation students (H1) nor influence career proficiency (H2) or career commitment (H3).

One related research area in which the issue of the psychological effect of emergencies matters is the measurement of the identity paradigm [37, 38]. The development and validation of vocational identity have long been the focus of vocational research pertaining to career exploration and career commitment [39, 40]. My Vocational Situation (MVS) is the most widely used measurement scale, tapping into an individual’s global awareness about their career goals and interests [41], whereas the Vocational Identity Measure (VIM) assesses how aware individuals are of their stable career goals, interests, and abilities [42].

Yet, only a handful of existing studies of vocational identity have extended to the employment aspirations and career behaviour of students or workers in the context of the pandemic [43]. Üngüren and Kaçmaz (2022) have acknowledged that the COVID-19 pandemic has triggered career anxiety in tourism students [44]. Another good case in point is the Australian aviation students, who are aware of the current oversupply of aviation professionals as a result of the industry downturn caused by the pandemic and are thereby looking for additional assistance with the development of non-technical skills in order to better prepare themselves to be competitive after graduation [16]. Similarly, final-year aviation students in Hong Kong want to widen their career prospects to include airline operations, aircraft engineering, and maintenance by acquiring new information and technical abilities during the pandemic [45]. In this respect, we argue that career proficiency might have some direct effect on employment aspiration (H4).

It has not escaped our notice that awareness of the hardships faced by medical professionals has increased motivation to pursue medicine as a career [46]. Similarly, airline workers’ perceived risks of virus infection and job instability significantly moderated the relationships among attitude, commitment, and employee career turnover intention [13]. Our point is that career commitment has a significant effect on the employment aspirations of Chinese aviation students (H5).

Above all, these growing bodies of research regarding risk perceptions of the pandemic can offer insight into the vocational identity and employment aspirations of Chinese aviation students in the context of the on-going pandemic. Accordingly, the SEM is used to measure the underlying relations between risk perceptions, employment aspirations, and vocational identity amidst the COVID-19 pandemic.

## Methods

### Participants

From August 2 to 8, 2022, an online survey was sent via the two most popular social networks in China, WeChat (with 1.1 billion monthly users) and QQ (with 0.73 billion monthly users), to Chinese students majoring in aviation service management who were under lockdown at six Chinese schools. These include the Sichuan TV and Film University, the Sichuan Aerospace Vocational College, and the Zhengzhou University of Aeronautics, the Urban Vocational College of Sichuan, the Sichuan Southwest Vocational College of Civil Aviation, and the Tianfu New General Aviation Profession Academy.

The study used a convenience-sampling approach to collect data and provide clear and consistent instructions to the respondents. The available pool of initial respondents was identified from the investigators’ WeChat and QQ lists, and they were encouraged to nominate another person with the same trait as the next subject. We promised that their answers would be used only for statistical purposes and remain strictly confidential and anonymous. Descriptive statistics were run to identify and exclude invalid samples. Occasional missing data on particular items were handled by a pair-wise deletion procedure. Of the total 629 surveys returned, 276 could be used for analysis.

### Measures

The survey instrument was a structured and self-administered questionnaire designed with the “Sojump”, an online survey platform (https://www.wjx.cn/vm/exhnoV9.aspx). Dillman’s Internet Survey Method was used to guide instrument design in order to increase the return rate [47].

The twelve items of COVID-19 pandemic perception were developed primarily from prior research into lay people’s views and responses to the pandemic, with additional items added to address the stringent Zero COVID policies [48]. Thus, respondents were asked to indicate their level of agreement on a five-point Likert scale from “1” (very disagreed) to “5” (very agreed) regarding their views of the pandemic and their responses to the COVID regulations in China. Vocational identity was assessed using eight questions tailored from either my vocational situation or the vocational identity measure by asking [49, 50]: “How do you view aviation jobs in the context of the strict COVID policies?” As to employment aspiration, respondents were asked to rate the possibility of choosing an aviation-related job after their graduation [51].

### Ethics

The data collection was conducted as part of a larger study focusing on the risk perceptions and career behaviours of Chinese students following the COVID-19 outbreak, which was run by the Chengdu University of Technology and did not require ethical board approval. The data were properly anonymized, and informed consent was furnished to respondents for review at the time of original data collection.

### Analysis

The Cronbach’s alpha test result for risk perceptions of the COVID-19 pandemic and vocational identity was 0.865 and 0.922, respectively. The statistical description was used to interpret the mean, valid frequency, and standard deviation of the dataset. Both explorative factor analysis (EFA) and confirmative factor analysis (CFA) were used to explore the data, because variables of vocational identity might be slightly different in dimensions from case to case and place to place, in particular amidst the COVID-19 Pandemic. Thus, the whole survey data was randomly split into two halves so as to avoid the subjectivity of data selection, one as a calibration sample (n = 140) and the other as a validation sample (n = 139). The dataset with the calibration sample was used to perform the EFA, while the other half was for the CFA. The overall model goodness of fit was reflected by the magnitude of discrepancy between the sample covariance matrix and the covariance matrix implied by the model with the parameter estimates. Model modification was required to obtain a better-fitting model. Once the model fitted well and was theoretically consistent, the parameter estimates and individual tests of significance were either to retain or reject the hypothesis.

## Results

### Profile of respondents

The demographical information of respondents was reported in Table 1. They mostly came from three schools, including the Sichuan Film and Television University (32.3%), the Sichuan Aerospace Vocational College (27.2%), and the Zhengzhou University of Aeronautics (29.0%). Most were female students (65.9%), either sophomores (61.3%) or juniors (31.5%) in the bachelor’s degree programme (70.3%). 68.5% of them were from the flight attendant programme, while around 30% were either from the aviation service and management (17.9%), the air guard (9.3%), or the ground services in the airport (4.3%).

**Table 1:** Demographics of the sample

	N		Percentage (%)		N	Percentage (%)
**Grade**				**Gender**		
2	171		61.3	Male	88	31.5
3	88		31.5	Female	184	65.9
4	20		7.2	* N/A*	7	2.5
* N/A*	0		0	**Age**		
**Program**				18 y or below	17	16.1
Advanced	196		70.3	19 y	110	39.4
Bachelor	83		29.7	20 y	93	33.3
* N/A*	0		0	21y	45	16.1
**School**				22y or above	14	5
Urban Vocational College of Sichuan	5		1.8	* N/A*	0	0
Sichuan TV and Film University	90		32.3	**Specialty**		
Sichuan Aerospace Vocational College	76		27.2	flight attendant	191	68.5
Sichuan Southwest Vocational College of Civil Aviation	20		7.2	air guard	26	9.3
Tianfu New General Aviation Profession Academy	7		2.5	aviation service and management	50	17.9
Zhengzhou University of Aeronautics	81		29	ground services	12	4.3
* N/A*	0		0	*N/A*	0	0

### Descriptive of risk perceptions and vocational identity

Respondents reported strong agreement on the perception of the COVID-19 pandemic (4.08 ≤ M ≤ 4.69). 93.9% strongly supported the stringent COVID policies, but over 80.0% felt worried about the current unstable situation, while 76.3% thought that people were refraining from outdoor activities. Over 90.0% thought that they had a good understanding of infection risk and the necessity of taking protective measures (see Table 2).

Respondents reported strong agreement on vocational identity (M = 3.99), because all eight variables were above the median on the Five-Point Likert scale (See Table 3). The four variables of career proficiency (0.815 ≤ SD ≤ 0.916), with a mean ranging from 4.15 to 4.23, were generally perceived as stronger than those of career commitment (3.67 ≤ M ≤ 3.90), but with a relatively higher standard deviation (1.001 ≤ SD ≤ 1.160).

**Table 2:** Descriptive statistics of risk perceptions of COVID-19 pandemic

Independent Variables	Mean	Standard Deviation	Valid percentage (%)				
			1	2	3	4	5
P1 I strongly support the stringent COVID-19 policies	4.69	0.623	0.4	0.4	5.4	17.6	76.3
P2 I am fully aware of the risk infected by COVID-19 patient	4.68	0.576	0.0	0.7	3.6	22.2	73.5
P3 I am fully aware of health measures to prevent infection	4.66	0.630	0.4	0.7	4.3	21.5	73.1
P4 I am fully aware of the risk infected by family members with COVID-19	4.65	0.678	0.7	1.1	3.9	21.5	72.8
P5 I am worried about the current situation under stringent approaches	4.52	0.795	1.1	1.1	9.3	21.9	66.7
P6 I am worried about people with illness	4.50	0.724	0.4	0.4	10.4	26.5	62.4
P7 I have sufficient knowledge of the stringent COVID-19 policies	4.45	0.717	0.0	0.7	11.1	30.5	57.7
P8 I am scared of being infected by the virus	4.42	0.826	0.7	1.1	14.3	23.7	60.2
P9 It is not safe to travel recently	4.30	0.857	0.7	1.8	16.5	29	52
P10 People refrain from out-door activities	4.15	0.996	2.2	4.3	17.2	29	47.3
P11 COVID-19 is more dangerous than SARS etc.	4.11	0.983	1.8	3.2	22.9	26.5	45.5
P12 I am worried about the long-lasting symptoms of COVID-19	4.08	1.044	1.8	6.5	20.4	24.4	47.0

It seems that most respondents stated that their abilities matched their work interests (M = 4.23; SD = 0.868). They could easily describe their ideal job to a recruiter of an aviation company (M = 4.15; SD = 0.856), had a clear sense of occupational interests in an aviation company (M = 4.18; SD = 0.91), and knew what career path in the aviation industry they wanted to pursue when they graduated from school (M = 4.20; SD = 0.815). Most of them had made a firm decision regarding what they wanted to do for a living (M = 3.90; SD = 1.159); they felt like they were on a definite aviation vocational path for the future (M = 3.86; SD = 1.001); but a few of them did not quite understand what type of aviation work they would like to do for the rest of their lives (M = 3.67; SD = 1.160); and they felt that the aviation vocation of their choice would be the best possible fit for them (M = 3.70; SD = 1.071).

### Factor analysis of personal health responses

The latent variables were suitable for structure detection (χ^2^ = 897.381, df = 28, p<0.001), as the KMO and Bartlett’s Test indicated over 93.9% of variance in the variables (KMO = 0.892) that might be caused by underlying factors (See Table 3). The rotation was converged in three iterations, indicating that two factors in the initial solution had eigenvalues greater than one, accounting for almost 79.074% of the variance (0.840>a>0.795).

**Table 3:** EFA and CFA of variables of vocational identity amidst the COVID-19

			Explorative Factor Analysis/EFA		Confirmative Factor Analysis /CFA		
	Mean /M	Standard Deviation/SD	Factor Loading		Squared Multiple Correlations /SMC	Standard Regression Weight/SRW	t-value/ C.R
**F**_**1**_ **Career Commitment**							
V5 I know what type of aviation work I would like to do for the rest of my life	3.67	1.160	0.725		0.637	0.798	6.207
V6 I have made a firm decision regarding what I want to do for a living	3.90	1.159	0.800		0.768	0.876	10.856
V7 I feel that the aviation vocation of my choice will be the best possible fit for me	3.70	1.071	0.901		0.539	0.734	8.980
V8 I feel like I am on a definite aviation vocational path for the future	3.86	1.001	0.821		0.287	0.536	6.207
**F**_**2**_ **Career Proficiency**							
V1My abilities match my work interests	4.23	0.868		0.860	0.537	0.733	10.354
V2 I know what occupational path in the aviation industry I want to pursue when I get out of school	4.20	0.815		0.854	0.732	0.855	14.077
V3 I have a clear sense of my occupational interests in an aviation company	4.18	0.916		0.744	0.686	0.828	9.556
V4 I could easily describe my ideal job to a recruiter of an aviation company	4.15	0.856		0.771	0.826	0.909	10.354
Eigen-value			5.367	0.959			
Explained Variance (%)			67.092	11.982			
Cumulative Explained Variance (%)			67.092	79.074			
Cronbach alpha Value			0.909	0.902			

The first factor was labelled “Career Commitment,“ which was most highly correlated with four variables, including “I know what type of aviation work I would like to do for the rest of my life” (V5), “I have made a firm decision regarding what I want to do for a living” (V6), “I feel that the aviation vocation of my choice will be the best possible fit for me” (V7), and “I feel like I am on a definite aviation vocational path for the future (V8). The second factor was named “Career Proficiency”, which was correlated with the other four variables, such as occupational interests and path.

### Structural model test

The dataset was adequate for CFA, with 79.1% of the variance explained by the two-factor solution (KMO = 0.892, χ^2^ = 897.381, df = 28, p<0.001). The overall measurement model with 10 observed variables, 14 unobserved variables, and 12 exogenous and 12 endogenous variables was formulated by combining the individual model of vocational identity with perceptions of the COVID-19 pandemic and employment aspirations.

Modification indices suggested to improve the model by introducing additional constraints or in such a way as to produce a relatively large increase in degrees of freedom, coupled with a relatively small increase in the chi-square statistic, which explained the theoretical reasons for suspecting that e_7 and e_8 might be correlated. Modification indices also suggested allowing some other error variables to be correlated, and these include e_1 and e_2, as well as e_10 and e_12. As a result, the goodness-of-fit indices for the overall measurement model indicated an acceptable level of fit between the model and the data (x^2^/df = 1.570, RMSEA = 0.064, TLI = 0.981, IFI = 0.993, CFI = 0.992). Within the overall model, the estimates of the structural coefficients provided the basis for testing the proposed hypotheses (See Table 4).

**Table 4:** Model fit assessment and modifications

	x2/df	CFI	TLI	RMSEA	PNFI	IFI	Model modifications
Model 1	4.606	0.869	0.803	0.162	0.561	0.871	
Model 2	4.491	0.869	0.810	0.159	0.579	0.871	G1<--C
Model 3	4.461	0.866	0.811	0.158	0.594	0.868	F2<---C
Model 4	1.371	0.986	0.980	0.052	0.655	0.986	e13<-->e14
Model 5	1.570	0.992	0.981	0.064	0.392	0.993	G<---F2; F1<---C

**Fig. 1:**
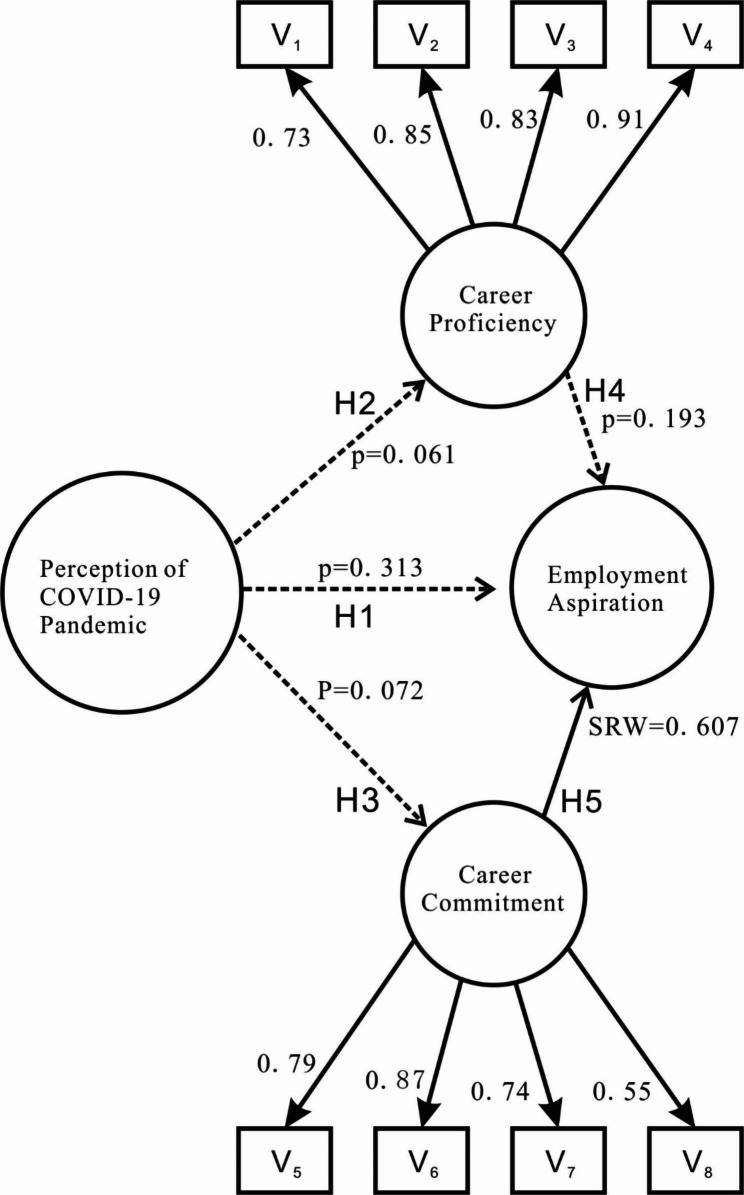
Modified structural equation model with an estimated path coefficient

Judging by the critical ratios (t critical value) and the standardized regression weights(0.536 ≤ SRW ≤ 0.869), only one of these five hypotheses would be accepted at conventional significance levels. The casual path from career commitment to employment aspiration (SRW = 0.607, t = 7.041) was supported (H5), while the regression weight for perception of pandemic in the prediction of career proficiency (p = 0.061) and commitment (p = 0.313) as well as employment aspiration (p = 0.072) was not significantly different from zero at the 0.001 level, suggesting that H1, H2, and H3 were fully supported. At the same time, the career proficiency was not significantly different from the mean of the employment aspiration (p = 0.191), failing to support H4 (See Fig. 1).

In other words, the structural equation modeling approach offered support for the statistically significant relationship between career commitment and employment aspirations of Chinese aviation students in the time of COVID-19, but career proficiency did not have any significant effect on employment aspiration. At the same time, sensitive perceptions of the pandemic failed to have any psychological effect on the two constructs of vocational identity and employment aspiration, demonstrating that the complexity of risk perceptions might lead to various types and degrees of personal responses towards epidemic threats.

## Conclusion and discussion

The COVID-19 crisis raises significant concerns for the entire education community: policy-makers, educators, parents, and learners. The purpose of this preliminary study is to report the risk perceptions, vocational identity, and employment aspirations of Chinese aviation students involved in China’s rigorous control measures of the pandemic. Of particular interest is to examine whether the pandemic has any effect on the employment aspirations of aviation students in China amidst the stringent Zero COVID policies.

One notable result is generated from contrasting perceptions of knowledge, awareness, and support for the stringent approach with worries and fears about the pandemic. Descriptive statistics suggest that respondents are generally sensitive to the pandemic. In spite of their strong support for the stringent Zero COVID policies and full awareness of infection risk and protective measures, they are worried about the current unstable situation and fear the severity and long lasting symptoms of COVID-19, which partly explains why students are likely to refrain from outdoor activities. Perhaps more jarringly, a very strong and sensitive perception of the pandemic fails to have any psychological effect on the two constructs of vocational identity and employment aspiration, similar to the findings that COVID-19 infection does not seem to affect cognition in college students [35]. In this case, poor risk communication might have resulted in overestimating or underestimating risk, sometimes with high infection risk-taking willingness [36], both of which would fail to have any effect on employment aspirations (H1), career proficiency (H2), and career commitment (H3).

To look further, the structural equation modeling approach offers support for the statistically significant relationship between career commitment and employment aspirations of Chinese aviation students in the time of the pandemic (H5), similar to medical professionals [52] and airline workers [13]. Yet, the argument that career proficiency might have some direct effect on employment aspiration (H4) is not supported.

From a practical point of view, the present study makes the following policy recommendations in response to the findings: To begin with, the situation that students possess insufficient COVID-19 knowledge and high-risk perceptions calls for further understanding the bounded rationality of college students so as to cope with anxiety disorder under lockdown [31, 36]. Secondly, the result here demonstrates the complexity of the public’s risk perceptions as “both feelings and analysis” that might lead to various types and degrees of personal responses towards epidemic threats [53]. Therefore, understanding risk perceptions of the pandemic may help guide on-going messaging to Chinese aviation students so as to be more psychologically resilient. Thirdly, it is not quite enough to worry about the current situation but to enhance their career proficiency and commitment, broaden their career options and adaptability, and have a clear career plan so that they may get well prepared for the fierce job market facing the next wave of the on-going pandemic, just as their counterparts do in Hong Kong and Australia [9, 16, 45].

In conclusion, this study will contribute to the emerging understanding of Chinese aviation students’ views and responses to the continuing threat of the COVID-19 pandemic in two ways: Firstly, the empirical findings of this study have identified the support for stringent COVID policies of Chinese aviation students and their awareness of infection risk and protective measures, which aligns with the knowledge, attitude, and practice model (KAP) among students in China, Egypt, and Indonesia [54, 55]. Secondly, the findings not only demonstrate the power of career commitment on employment aspirations but also reveal that a relatively high self-assessment of career proficiency may not necessarily lead to a clear career aspiration, possibly due to poor risk communication and insufficient career planning guidance. This situation is contrary to the previous findings in Hong Kong and Australia, where aviation students perceived themselves as lacking technical skills and were interested in learning more knowledge and skills or broadening their career options [16, 45].

The preliminary results presented here need to be qualified in at least three different ways: Firstly, it will be useful to include other variables related to public perceptions in a future study, such as information sources and preferences, travel intention and behaviour, as well as cognitive and emotional experiences. Secondly, the argument that respondents possess insufficient COVID-19 knowledge but high infection risk perceptions needs to be further verified in line with the KAP. We do have interest in understanding the question of whether Chinese students are truly familiar with the zero-COVID policies, apart from the objective of achieving zero mortality rates, especially those policies relevant to the aviation industry. Yet, due to the limitation of the research design, in particular instrument design, we could not fully understand the students’ knowledge of the stringent COVID-19 policies and its sustaining impacts on the aviation industry. Thirdly, a limited number of online surveys with certain subjectivity were mostly collected from vocational schools in Sichuan amidst the stringent COVID policies declared by the Chinese health authority. Understanding and attitude of the dynamic zero-COVID policy may affect their responses to politically sensitive questions, challenging the reliability of the survey. We argue that it is detrimental for students who have not fully understood the long-lasting and devastating effects that the pandemic is having on the low employment of the Chinese aviation industry [56]. This is evidenced by the fact that the COVID-19 has resulted in a dramatic drop in demand for airline services at a time when many countries have reduced international flights and closed borders to China, creating an uncertain perspective for aviation students facing the multi-wave of infectious risk. As risk, fear, and uncertainty continue, the psychological effects of this ongoing virus truly deserve concern from both spatial and temporal perspectives. Thus, the respondents might be targeted in person and further expanded to schools domestically as China has adjusted its dynamic strategy for COVID-19 prevention and control in recent months, which lays the foundation for its reopening.

## Data Availability

The datasets generated and/or analysed during the current study are not publicly available due to this type of use not being included in the written consent form but are available from the corresponding author on reasonable request.

## References

[1] Lin QY, Zhao QLS, Gao D, Lou Y, Yang S, Musa SS (2020). A conceptual model for the coronavirus Disease 2019 (COVID-19) outbreak in Wuhan, China with individual reaction and governmental action. Int J Infect Dis.

[2] Cai JC, Hu SY, Lin QY, Tan R, Chen LB (2022). China’s ‘dynamic zero COVID-19 strategy’ will face greater challenges in the future. J Infect.

[3] Tan CQ, Luo X, Zhou Z, Zeng XH, Wan XM, Yi LD (2023). Dynamic zero-COVID strategy in controlling COVID-19 in Shanghai, China: a cost-effectiveness analysis. J Infect Pubic Health.

[4] Bai L, Lu HN, Hu HL, Smith MK, Harripersaud K, Lipkova V (2021). Evaluation of work resumption strategies after COVID-19 reopening in the Chinese city of Shenzhen: a mathematical modeling study. Pubic Health.

[5] Wang Q, Huang R (2021). The impact of COVID-19 pandemic on sustainable development goals – a survey. Environ Res.

[6] Jin H, Kong QX, Wang HM (2020). COVID-19 prevention and control strategy: management of close contacts in Hangzhou City, China. J Infect Pubic Health.

[7] Su M, Hu BY, Liu WX, Tian C (2022). Effects of COVID-19 on China’s civil aviation passenger transport market. Res Transp Econ.

[8] Warnock-Smith D, Graham A, O’Connell JF, Efthymiou M (2021). Impact of COVID-19 on air transport passenger markets: examining evidence from the Chinese market. J Air Transp Manag.

[9] Li YL, Wang JO, Huang J, Chen Z (2022). Impact of COVID-19 on domestic air transportation in China. Transp Policy.

[10] Wandelt S, Sun XQ, Zhang AM (2023). On the contagion leakage via incoming flights during China’s aviation policies in the fight against COVID-19. J Air Transp Manag.

[11] Liu AY, Kim YR, O’Connell JF (2021). COVID-19 and the aviation industry: the interrelationship between the spread of the COVID-19 pandemic and the frequency of flights on the EU market. Ann Tour Res.

[12] Yu M, Chen ZH (2021). The effect of aviation responses to the control of imported COVID-19 cases. J Air Transp Manag.

[13] Han H, Koo B, Ariza-Montes A, Lee Y, Kim HR (2021). Are airline workers planning career turnover in a post-COVID-19 world? Assessing the impact of risk perception about virus Infection and job instability. J Hosp Tour Manag.

[14] Sun XQ, Wandelt S, Zhang A (2023). A data-driven analysis of the aviation recovery from the COVID-19 pandemic. J Air Transp Manag.

[15] Li JL, Che WY (2022). Challenges and coping strategies of online learning for college students in the context of COVID-19: a survey of Chinese universities. Sustain Cities Soc.

[16] Miani P, Kille T, Lee YS, Zhang YH, Bates RP (2021). The impact of the COVID-19 pandemic on current tertiary aviation education and future careers: students’ perspective. J Air Transp Manag.

[17] CAAC. 2022. 2021 annual report on civil aviation industry in China. Retrieved from.http://www.caac.gov.cn/XXGK/XXGK/TJSJ/202205/t20220518_213297.html.

[18] Azhar IE, Hui DCS, Memish AZ, Drosten C, Zumla A (2019). The Middle East Respiratory Syndrome (MERS). Infect Dis Clin North Am.

[19] Cherry CC, Beer DK, Fulton C, Wong D, Buttke D, Staples EJ (2016). Knowledge and use of prevention measures for Chikungunya virus among visitors - Virgin Islands National Park. Trav Med Infect Dis.

[20] Cahyanto I, Wiblishauser M, Pennington-Gray L, Schroeder A (2016). The dynamics of travel avoidance: the case of Ebola in the U.S. Tour Manag Perspect.

[21] Park JJH, Mogg R, Smith GE, Nakimuli-Mpungu E, Jehan F, Rayner CR (2021). How COVID-19 has fundamentally changed clinical research in global health. Lancet Glob Health.

[22] Wang Y, Di Y, Ye J, Wei W (2020). Study on the public psychological states and its related factors during the outbreak of coronavirus Disease 2019 (COVID-19) in some regions of China. Psychol Health & Med.

[23] Vijayalakshmi P, Kathyayani VB, Sreelatha M, Reddy S, Manjunatha N, Kumar NC (2023). Resilience as a protective factor on the quality of life (QoL) of Indian nursing students during the COVID-19 pandemic. Arch Psychiatr Nurs.

[24] Zhang YX, Geddes J, Kanga JG, Himelhoch S (2022). Psychological impacts of the COVID-19 pandemic on medical students in the United States of America. Arch Psychiatr Res.

[25] Czerny AI, Fu XW, Lei Z, Oum TH (2021). Post pandemic aviation market recovery: experience and lessons from China. J Air Transp Manage.

[26] Linden E (2021). Pandemics and environmental shocks: what aviation managers should learn from COVID-19 for long-term planning. J Air Transp Manage.

[27] Dryhurst S, Schneider CR, Kerr J, Freeman ALJ, Recchia G, van der Bles AM (2020). Risk perceptions of COVID-19 around the world. J Risk Res.

[28] Schneider CR, Dryhurst S, Kerr J, Freeman ALJ, Recchia G, Spiegelhalter D (2021). COVID-19 risk perception: a longitudinal analysis of its predictors and associations with health protective behaviours in the United Kingdom. J Risk Res.

[29] Ye B, Chen X, Zhang YZ, Yang Q (2022). Psychological flexibility and COVID-19 burnout in Chinese college students: a moderated mediation model. J Contextual Behav Sci.

[30] Cao W, Fang Z, Hou G, Han M, Xu X, Dong J (2020). The psychological impact of the COVID-19 epidemic on college students in China. Psychiatry Res.

[31] Jie F, Lau PWC, Lei S, Huang WY (2022). Movement behaviors and post-traumatic stress disorder during the COVID-19 pandemic: a retrospective study of Chinese university students. J Exerc Sci Fit.

[32] Ma H, Miller C (2020). Trapped in a double bind: Chinese overseas student anxiety during the COVID-19 pandemic. Health Commun.

[33] Seehuus M, Stanton AM, Handy AB, Haik AK, Gorman R, Clifton J (2021). Impact of COVID-19 predicts perceived risk more strongly than known demographic risk factors. J Psychosom Res.

[34] Akritidis J, McGuinness SL, Leder K (2023). University students’ travel risk perceptions and risk-taking willingness during the COVID-19 pandemic: a cross-sectional study. Travel Med Infect Dis.

[35] Francis G, Thunell E (2023). COVID-19 Infection does not seem to affect cognition in college students. Conscious Cogn.

[36] Jiang RC (2020). Knowledge, attitudes and mental health of university students during the COVID-19 pandemic in China. Child Youth Serv Rev.

[37] Sampson JP, Peterson GW, Reardon RC, Lenz JG (2000). Using readiness assessment to improve career services: a cognitive information processing approach. Career Dev Q.

[38] Multon KD, Wood R, Heppner MJ (2007). Gysbers NC.A Cluster-analytic investigation of subtypes of adult career counseling clients: toward a taxonomy of career problems. J Career Assess.

[39] Holland J, Johnston J, Asama F (1993). The Vocational Identity Scale: a diagnostic and treatment tool. J Carrer Assess.

[40] Marcia JE (1966). Development and validation of ego-identity status. J Pers Soc Psychol.

[41] Holland J, Gottfredson DC, Power PG (1980). Some diagnostic scales for research in decision making and personality: identity, information, and barriers. J Pers Soc Psychol.

[42] Gupta A, Chong SH, Leong FTL (2014). Development and validation of the vocational identity measure. J Career Assess.

[43] Keijzer R, van der Rijst R, van Schooten E, Admiraal W (2021). Individual differences among at-risk students changing the relationship between resilience and vocational identity. Int J Educ Res.

[44] Üngüren E, Kaçmaz YY (2022). Does COVID-19 pandemic trigger career anxiety in tourism students? Exploring the role of psychological resilience. J Hosp Leis Sport Tour Educ.

[45] Yui CY, Ng KKH, Yu SGM, Yu CW (2022). Sustaining aviation workforce after the pandemic: evidence from Hong Kong aviation students toward skills, specialised training, and career prospects through a mixed-method approach. Transp Policy.

[46] Saleh R, Martins RS, Saad M, Fatimi AS, Kumar G, Abbas M (2022). The impact of the COVID-19 pandemic on the career choice of medicine: a cross-sectional study amongst pre-medical students in Pakistan. Ann Med Surg.

[47] Dillman DA, Smyth JD, Christian LM. Internet, mail, and mixed-mode surveys: the tailored design method. NY Wiley; 2009.

[48] Tang Y, Wang YR, Liang Y (2021). Lay people’s view and responses to the pandemic: perceptions of COVID-19 and personal health responses in China. Asian Pac J Pub Health.

[49] Holland J (1996). Exploring careers with a typology: what we have learned and some new directions. Am Psychol.

[50] Li X, Hou ZJ, Jia Y (2015). The influence of social comparison on career decision-making: vocational identity as a moderator and regret as a mediator. J Vocat Behav.

[51] Hirschi A, Herrmann A (2013). Assessing difficulties in career decision making among Swiss adolescents with the German my vocational situation scale. Swiss J Psychol.

[52] Saefi M, Fauzi A, Kristiana E, Adi WC, Muchson M, Setiawan ME (2020). Survey data of COVID-19-related knowledge, attitude, and practices among Indonesian undergraduate students. Data Brief.

[53] Slovic P (1987). Perception of risk. Sci.

[54] Xue Q, Xie XY, Liu Q, Zhou Y, Zhu KH, Wu H (2021). Knowledge, attitudes, and practices towards COVID-19 among primary school students in Hubei Province, China. Child Youth Serv Rev.

[55] Salem MR, Hanafy SHA, Bayad AT, Abdel-aziz SB, Shaheen D, Amin TT (2021). Assessment of knowledge, attitudes, and precautionary actions against COVID-19 among medical students in Egypt. J Infect Public Health.

[56] Serrano F, Kazda A (2020). The future of airports post COVID-19. J Air Transp Manag.

